# Histological and Demographic Characteristics of the Distribution of Brain and Central Nervous System Tumors’ Sizes: Results from SEER Registries Using Statistical Methods

**Published:** 2012-09

**Authors:** Keshav P. Pokhrel, Dimitrios Vovoras, Chris P. Tsokos

**Affiliations:** *Department of Mathematics and Statistics, University of South Florida, USA*

**Keywords:** brain and CNS, parametric analysis, prognostic variables, quantile regression, SEER, survival rate, tumor size

## Abstract

The examination of brain tumor growth and its variability among cancer patients is an important aspect of epidemiologic and medical data. Several studies for tumors of brain interpreted descriptive data, in this study we perform inference in the extent possible, suggesting possible explanations for the differentiation in the survival rates apparent in the epidemiologic data. Population based information from nine registries in the USA are classified with respect to age, gender, race and tumor histology to study tumor size variation. The Weibull and Dagum distributions are fitted to the highly skewed tumor sizes distributions, the parametric analysis of the tumor sizes showed significant differentiation between sexes, increased skewness for both the male and female populations, as well as decreased kurtosis for the black female population. The effect of population characteristics on the distribution of tumor sizes is estimated by quantile regression model and then compared with the ordinary least squares results. The higher quantiles of the distribution of tumor sizes for whites are significantly higher than those of other races. Our model predicted that the effect of age in the lower quantiles of the tumor sizes distribution is negative given the variables race and sex. We apply probability and regression models to explore the effects of demographic and histology types and observe significant racial and gender differences in the form of the distributions. Efforts are made to link tumor size data with available survival rates in relation to other prognostic variables.

## INTRODUCTION

The importance of epidemiologic and medical data, and inference on the extent possible for primary brain tumors and tumors of the Central Nervous System (CNS) has been previously recognized (1, 2). Relevant rates and prognostic information come primarily from clinical trials and population registry data. Clinical trials usually provide more complete information on prognostic factors, since one pathology has been reviewed as a whole. On the other hand, estimates based on population registry data are reflecting a bigger picture of patients but with considerably larger variance for the times and types of diagnoses.

Many studies for tumors of the brain and CNS have examined and interpreted descriptive and epidemiologic data suggesting possible explanations for the changes in the disease rates. Brain tumor growth and its differentiations account for some of the variability in the survival rates emphasizing the importance of tumor size in the prognosis of patients with brain tumor (3, 4). The statistics reflected in this study represent a significant portion of the US population and the data is maintained with high standards for different geographical and ethnic populations, supporting our central goal to report what may possibly make the brain and CNS an undesirable environment for tumor progression.

For this study, we select the size of the tumor to be the variable of interest for different types of tumor and demographic characteristics. The distribution of tumor types with age is reported by the Central Brain Tumor Registry of the United States (CBTRUS). Higher incidence rates in males than in females for most histologies have been reported in (5), as well as racial differences in occurrence rates. Trends in incidence and survival in the United States have been studied in (6), increased risk of brain cancer was associated with being male, Caucausian, elderly. In (7) and (8) cancer survival trends are evaluated and changes in the survival rates suggest possible explanations for the improved prognosis. Finally, prevalence rates for the US population are studied in (9).

We aim to present our findings with regard to diagnosed brain tumors in the USA from 1973 up to 2006. The National Cancer Institute’s Surveillance, Epidemiology and End Results (SEER) program provided us with information for malignant brain tumors. To better understand the relationship between demographic characteristics and the brain tumor prognosis we identified the probability distributions that best describe the variability of the tumor size records for different races and sexes. Such a characterization is essential in order to obtain information about the central tendency, variance and skewness of distributions specific to race and sex and postulated about their effect on the tumor size. Furthermore, in an attempt to better understand the role of age on the tumor size, we study its effect on the distribution of tumor sizes in the presence of two other predictors, namely race and gender and considered all possible interactions as potential prognostic factors for the tumors’ variation.

## METHODS

The discussion below presents the data from several cancer registries in the United States of America, as well as the complete compilation of various brain tumor types included in the study. Finally, we tabulate the distribution of tumor types with respect to age groups, race and gender.

### Selection and Description of Participants

The Surveillance, Epidemiology and End Results program is a comprehensive source of population based information on cancer registries covering 28% of the population in the USA collecting complete and accurate data on all cancers diagnosed. SEER periodically report incidence, mortality and survival data as well as the extend of disease at diagnosis and link those with other national data sources to identify unusual changes and differences in the patterns. Specific goals of SEER include the facilitation of collaboration among the scientific community and the encouragement in use of surveillance data from researchers, public health officials, policy makers and the public for cancer prevention, monitoring and control interventions.

In this study we analyze data on malignant primary brain tumors diagnosed from 1973 through 2006 which are available for the following registries: Atlanta, Connecticut, Detroit, Hawaii, Iowa, New Mexico, San Francisco-Oakland, Seattle-Puget Sound, and Utah, Los Angeles, San Jose-Monterey, Rural Georgia, and the Alaska Native Tumor Registry, Greater California, Kentucky, Louisiana, and New Jersey. The data set includes 72,770 primary malignant brain tumor cases: 37,150 males and 35,620 females.

The information for the histology of the brain tumors provided by SEER includes definitions for cancer morphology and topography and is based in the ICD-0-3 histology (10). Primary site codes are C000-C809 and ICD-O-3 histology codes are 9161-9571 as well as 8000-8005 finally, 0, 1 and 3 are ICD-O-3 behavior recodes. Tumors vary greatly in size and positions, the shape is also inevitably subjective and becomes infeasible in large datasets. SEER records for primary tumors were measured as the largest dimension or diameter of the tumor in mm’s. The research data we are interested included 11,331 male (87.6% white, 5.8% black, 6.6% other races) and 11,027 female (84.7% white, 7.5% black, 7.8% other races) individuals with recorded tumor sizes.

### Technical Information

The classification of tumors for adolescent and young adults (AYA) was developed to better understand major cancer sites and facilitate the reporting of cancer incidence rates and trends. The histological site groups for the tumors that are used in the SEER have been based on the classification scheme proposed in (11) for cancer morphology and topography; six main diagnostic groups are defined, half of them have subgroups of two or three members. It is mentioned in the paper cited before that “Included in the considerations at that time were the desirability of having a standard framework while allowing for the flexibility of subdivisions within a small number of main groups, and the allocation of the maximum number of codes to specific categories so that the number of malignancies grouped as “other” is minimized.”

**Figure 1:**
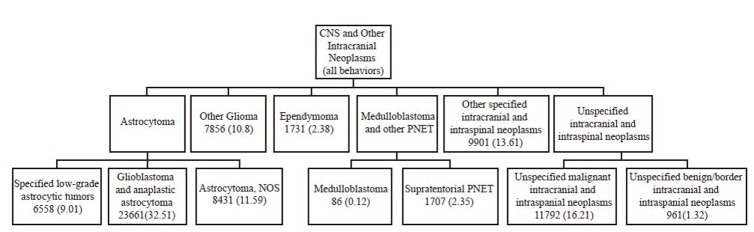
The above classification scheme for brain tumors is based on the classification scheme proposed by RD Barr and colleagues. The variables were updated from the original ICD-O-2 based classification scheme using ICD-O-3 definitions for cancer morphology and topography.

The groups are further delineated for more detailed analysis of the information in terms of specific histological subgroups. Astrocytoma, is subdivided in specified low grade astrocytic tumor, glioblastoma and anaplastic astrocytomas, astrocytoma non-otherwise specified (NOS). Other glioma, ependymoma, medulloblastoma and other primitive neuroectodermal tumors (PNET) subdivided in medulloblastoma, supratentorial PNET. Finally, other specified intracranial and intraspinal neoplasms, unspecified intracranial and intraspinal neoplasms subdivided in unspecified malignant intracranial and intra spinal neoplasm and unspecified benign/boarder intracranial and intraspinal neoplasms, the classification scheme is presented in Figure 1.

Descriptive information for the total number of tumors by race, sex, histology is presented in Table 2, the numbers shown at the body of the table refer to percentages of the diagnosed population out of the total numbers found on the right column. Cases are racially classified as white or nonwhite. The most frequently reported histologies are Glioblastoma and anaplastic astrocytoma (32.5%), Unspecified malignant intracranial intraspinal neoplasms (16.2%) andother specified intracranial and intraspinal neoplasms (13.6%) which account for over one half of the reported tumors. A large number of astrocytomas (21.8%) is classified as non-otherwise specified. Age distributions differ by histology type suggesting different etiologic factors are active. Medulloblastic tumors are more prevalent in children, 69.7% of medulloblastoma patients are diagnosed before the age of 20 and more common among males (12). Finally, ependymoma is more frequent in females than males, for a comprehensive analysis of rates and their time trends see (13).

For the parametric analysis of the tumor sizes, the probability distributions that best fit the tumor sizes data are the Dagum or inverse Burr, widely used to describe the distribution of personal income. The Dagum distribution is closely related to the generalized beta II distribution and can be parameterized either with three or four parameters (14) as shown in equation (a). Also, the Weibull probability distribution frequently used in reliability engineering (15), characterizes tumor sizes for females in most of the cases, the analytical representation is shown in equation (b). The probability distributions are selected here using random samples of 5000 from the data at hand; the Kolmogorov Smirnov goodness of fit test applied for the identified distributions.

(a) $$\begin{array}{cc} \frac{\text{Probability}\;\text{density}\;\text{function}}{f\left(x\right)=\frac{\alpha k{\left(\frac{x-\gamma }{\beta }\right)}^{\alpha k-1}}{\beta {\left(1+{\left(\frac{x-\gamma }{\beta }\right)}^{\alpha }\right)}^{k+1}}} & \frac{\text{Cummulative}\;\text{density}\;\text{function}}{F\left(x\right)={\left(1+{\left(\frac{x-\gamma }{\beta }\right)}^{-\alpha }\right)}^{-k}} \end{array}$$

(b) $$\begin{array}{cc} g\left(x\right)=(\frac{\alpha }{\beta }){\left(\frac{x}{\beta }\right)}^{\alpha -1}\exp\left(-{\left(\frac{x}{\beta }\right)}^{\alpha }\right) & G\left(x\right)=1-\exp\left(-{\left(\frac{x}{\beta }\right)}^{\alpha }\right) \end{array}$$

In the case of the Dagum four parameter distribution α, k are shape parameters, β is the scale parameter finally, γ is the location parameter. When γ=0 resulting in the (0, ∞) domain for the probability distribution we refer to the three parameters Dagum distribution. For the Weibull distributionα, β are the shape and scale parameters respectively. The product α•k for the paramemeters of the Dagum distribution measures the rate of increase from zero for x→0, or the probability mass of the tail (14) and is larger in the male than the female in the only relevant case, resulting in a greater probability mass in the case of females for the left tail. We list the maximum likelihood estimates of the identified distributional parameters in Table 1.

**Table 1:** The identified probability distributions along with the estimates for the corresponding parameters that best fit the tumor sizes data

	Males	Females

All races	Dagum (4p): *k*=0.36, *α*=5.45, *β*=55.78, *γ*=0.06	Weibull: *α*=2.02, *β*=43.51
White	Dagum (4p): *k*=0.35, *α*=5.65, *β*=56.47, *γ*=0.10	Weibull: *α*=2.09, *β*=43.98
Black	Dagum (3p): *k*=0.45, *α*=4.31, *β*=54.54	Dagum (4p): *k*=0.47, *α*=3.68, *β*=45.43, *γ*=0.41
Other	Dagum (4p): *k*=0.36, *α*=5.17, *β*=57.57, *γ*=0.37	Weibull: *α*=1.89, *β*=44.13

**Table 2:** Characteristics of the study population by histology type, race, sex, the numbers shown at the body of the table refer to percentages of the diagnosed population

Histology	Age category			Race/Gender			Total
	0-19	20-64	65+	White male	Non-white male	White female	

Astrocytoma							
Specified low grade astrocytic tumor	9.1	43.8	46.9	45.3	6.2	42.3	6558
Glioblastoma and anaplastic astrocytoma	9.6	47.0	43.3	46.6	5.6	42.9	23661
Astrocytoma NOS	9.8	43.6	46.5	42.8	9.2	37.9	8431
Other Glioma	10.0	43.6	45.8	45.4	7.2	40.8	7856
Ependynoma	5.8	52.1	42.0	30.9	9	43.8	1731
Medulloblastoma and other Primitive Neuroectodermal Tumors (PNET)							
Medulloblastoma	69.7	26.7	3.4	48.8	3.5	36	86
Supratentorial PNET	6.5	45.2	48.2	50.4	3.2	43.5	1707
Other specified intracranial and intraspinal neoplasms	9.0	51.7	39.2	37.4	7.9	40.8	9901
Unspecified intracranial and intraspinal neoplasms							
Unspecified malignant intracranial intraspinal neoplasms	11.7	52.3	35.9	50.4	4.2	41.8	11792
Unspecified benign intracranial intraspinal neoplasm	3.1	58.3	38.5	30.8	4.9	57.5	961
Total	9.3	46.3	43.8	44.8	6.2	42.5	72770

The sums of the subgroups listed donot equal the total. Tumor types included in the total may not be in a specific subgroup of SEER classification.

## RESULTS

In the following subsections we summarize brain tumor sizes by sex, age, race and gender for patients with different tumor types. Significantly different mean tumor sizes for histology specific tumors may have important implications. Finally, we report the differences in the probability distributions of the tumor sizes fitted in population subgroups.

### Comparisons of Mean Tumor sizes

Age specific average tumor sizes by sex are plotted to present the variability for various ages. For specific histologies and age groups we compute the mean tumor size and statistically compare gender specific averages. Taking into consideration the complexity of the data we performed all pairwise comparisons non-parametrically, not relying in any particular distribution. In the subsequent analysis we have disregarded tumors with sizes more than 105 mm, those tumors were deemed to challenge the robustness of our analysis.

**Figure 2:**
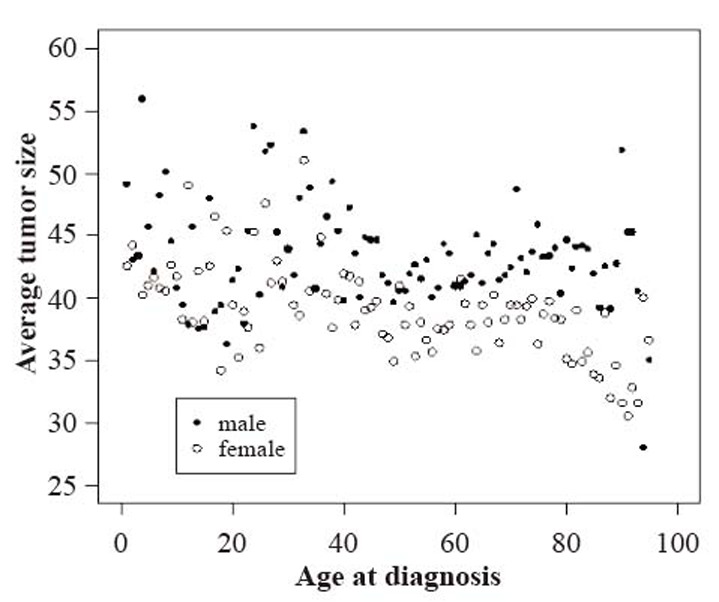
Average tumor size (the largest dimension or diameter of the primary tumor in mm) against age at diagnosis for tumors diagnosed from 1973-2006 included in the SEER 9 registries database. Average tumor sizes reported for males are shown with bold circles and a hollow circle is used for female records.

A standardized system for the analysis and presentation of data regarding tumors diagnosed in a specific age group for the given classification will greatly facilitate comparisons of interest and generate interesting hypotheses. To this end we plot the annual mean tumor sizes for males and females and comment on the behavior for different data driven age groups in Figure 2. The different age categories evident here are in accordance with the scheme that is followed in most of the reports in the literature.

Evidently, average sizes of tumors diagnosed before forty years of age exhibit greater variability than those tumors diagnosed later and this behavior is common in both sexes. The data is less volatile for tumor sizes diagnosed in individuals between the ages of forty and seventy, though the tumor sizes are consistently larger for males.

For the ages less than forty there is a distinction in the behavior between different sexes, the male records exhibit a concave up behavior with a peak at the age of twenty, while the female records do not present any clear pattern. Lastly, for the ages above forty male tumor sizes do not show any clear pattern while female tumors clearly present a downward trend. We note that tumor sizes for the ages above seventy-five were computed with less data compared to other ages.

Considering the importance of studying mean tumor sizes we further delineate the data by histology and gender. The vast majority of people in the data set were white (87.3%) whereas diagnosed tumors in blacks comprised 7.1% of the total number of patients. About 9.4% of the tumors occurred in children (0-19 years old), whereas 46.3% of them were in the 20 to 64 age group, with the remaining 43.9% occurring in the elderly (65 years or older). The 36 histology specific sample means for three age groups and sexes along with the corresponding sample sizes are tabulated in Table 1.

For the six histologies combined, the mean tumor size for males is larger than that for females (*p*-value<0.00005). We tested the equality of mean tumor sizes betweeen males and females for specific histologies; all comparisons were made using the Kruskal-Wallis test. Only in the case of medulloblastoma we failed to reject the null hypothesis (*p*-value=0.42), the mean tumor size of medulloblastic tumors does not differ. When testing the same hypothesis for the mean tumor size in the three different racial groups we failed to reject in all cases. The tabulated average tumor sizes followed by total number of patients in parentheses, refer to patients diagnosed with malignant brain tumors between 1969 and 2006.

### Parametric analysis

The main advantage of parametric methodology is that the information contained in the very large data sets can be concentrated in a small number of parameters. Furthermore, useful information can be drawn directly from the estimated parameters for different subpopulations, provided that the differences in the estimated parameters have a clear biological interpretation. Therefore, one of the undisputed properties required for the probabilistic models of distributions of tumor sizes is their biological interpretation. To formulate the analysis we have partitioned the data with regard to race into whites, blacks and a third class containing the remaining races as well as gender of the patient.

**Figure 3:**
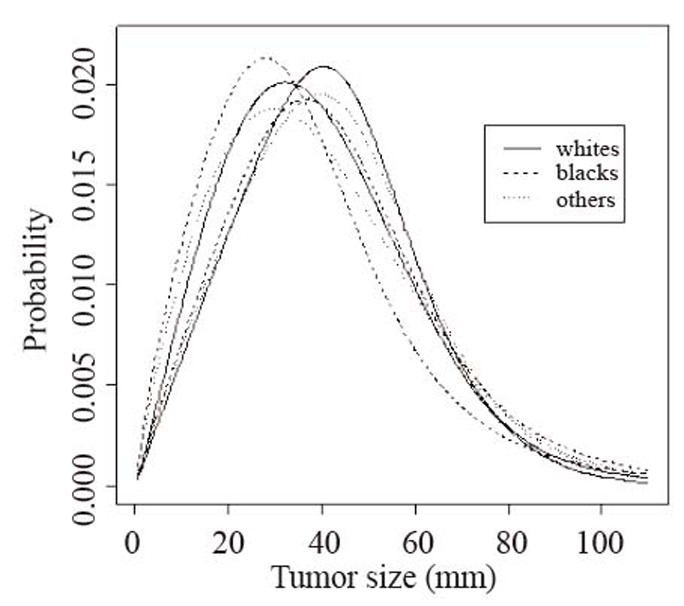
Plots of fitted probability distribution functions (PDF) of tumor sizes classified for gender and race. The leftmost triad of curves corresponds to PDF’s for females and the rightmost triad of curves corresponds to males. The identified probability distributions along with the estimates for the corresponding parameters that best fit the tumor sizes data are shown below.

In Figure 3, we plot below the identified probability density curves that best characterize the tumor sizes for six racial/gender subpopulations. The basic characteristics of the tumor sizes for those subgroups are reported in Table 4, we can initially note a clear distinction between the distributions of male and female tumor sizes, the modes of the respective distributions of tumor sizes are around 40 mm and 27 mm. In further detail, the probability distribution for the black females is more skewed compared to any other of the identified distributions.

Having knowledge of the probability distributions we can have a better understanding of the tumor sizes variability and estimate basic measures for the tumor records in different population subgroups. In the following we tabulate the means, medians, variances, skewnesses measure of symmetry, and kurtoses, measure of flatness. We are using the estimated values of the appropriate distributional parameters to compare the basic characteristics of the tumor sizes for the populations of interest (the mathematical formulas used to calculate the statistics of Table 3 are presented in Appendix A).

**Table 3:** Average tumor sizes (in mm) by major histological type for primary brain tumors across three different age groups (0-19, 20-64, >65 years of age) with corresponding number of diagnosed cases in *italics*

Histology	Age Groups (Males)			Age Groups (Females)		
	0-19	20-64	65+	0-19	20-64	65+

Astrocytoma	43.3 5(*704*)	43.36 (*3328*)	42.91 (*2096*)	42.82 (517)	39.75 (*2805*)	38.22 (*2305*)
Other Glioma	42.27 (*222*)	44.37 (*867*)	45.17 (*562*)	46.42 (*168*)	42.67 (*617*)	40.69 (*605*)
Ependymoma	41.35 (*23*)	41.91 (*257*)	41.56 (*130*)	39.56 (*18*)	35.95 (*326*)	28.11 (*307*)
Medulloblastoma	43.86 (*70*)	46.13 (*287*)	46.02 (*159*)	40.70 (*47*)	48.72 (*176*)	46.04 (*141*)
Other Specified	47.33 (*209*)	41.56 (*1274*)	41.00 (546)	42.86 (1*88*)	35.81 (*1275)*	34.89 (878)
Unspecified	42.22 (*90*)	43.01 (*655*)	45.03 (*510*)	36.23 (*77*)	39.58 (*574*)	40.21 (*561*)

From Table 4, the difference between the mean and median in the black populations is about double the corresponding difference in the other populations. Also, skewness for both the black male and black female populations is larger than other subgroups, that is, on average there is a higher number of tumor sizes more distant from the median. Although as we have already mentioned, the black comprise only 7.8% of the individuals in the data set, there is strong evidence that the variance is largest in the black subpopulation.

**Table 4:** Estimates of central tendency and variability for tumor sizes classified according to race and gender were obtained using estimated parameters for the identified probability distributions shown under Figure 3

	Median/Mean		Variance		Skewness		Kurtosis	
	male	female	male	female	male	female	Male	female

All races	40.38/42.36	36.29/38.55	457.10	398.68	1.35	0.62	11.30	0.02
White	40.93/42.65	36.90/38.95	442.18	383.06	1.22	0.58	9.75	0.01
Black	40.35/44.22	33.06/37.84	692.22	707.71	2.69	4.87	68.67	-92.03
Other	41.27/43.62	36.35/39.17	533.25	464.14	1.54	0.71	14.15	0.04

Measuring the size of kurtosis observed in the data sets, we find out that the value for the black male individuals is three times as big as the second largest. Higher kurtosis means more of the variance is a result of infrequent extreme deviations from the mean as opposed to frequent modestly sized deviations. It is worth noting that, on the other extreme, tumor sizes for black females contain the least amount of extreme observations from the mean compared to all other data sets.

To our knowledge there has been no systematic analysis of the brain tumor sizes and differences associated with the effect of the race and gender on the respective distributions (the Surveillance Epidemiology and End Results organization does not analyze data on tumor size because of the large amount of missing data). Such measurements though are commonly used in the evaluation of diagnosed tumors and have implications for patient prognosis and treatment. In Table 5 we report probabilities of different tumor sizes for male and female patients based on the parameter estimates of the fitted distributions. The 5-year relative survival rates related to the tumor size characterization can be found (16) on the last two columns.

**Table 5:** Cancer of the Brain and other CNS: Sex specific probabilities and relative survival rates for different tumor sizes

Tumor size	Probability		Relative survival rate 5 year (%)	
	Male	Female	Brain	Other CNS

<20 mm	0.133	0.18	31.5	89.2
20-50 mm	0.555	0.546	19.8	71.2
>50 mm	0.311	0.266	20.8	58.3

It is reported in (16) that for diagnosed brain tumor sizes ≤20 mm, 20-50 mm, >50 mm the 5-year relative survival rates are 31.5, 19.8, 20.8, respectively and 89.2, 71.2, 58.3, respectively for tumors of the other central nervous system. The 5 year relative survival rates are not significantly different for patients with tumor sizes between 20-50 mm and bigger than 50 mm. Survival differences in subpopulations can be associated to the differences in the distributions of tumor sizes. In the following section we will attempt to model the effect of those covariates on the quantiles of the distribution of tumor sizes.

### Quantile Regression Model

To be able to measure the effect of population characteristics on the tumor size in even more detail we employ regression models for different quantiles of the distribution of tumor sizes. We begin this section with a brief introduction to the model, and then immediately apply it to our dataset. Standard least squares regression models calculate the average effect of the independent variables on the tumor size. However (17), the focus on the average tumor size may hide important elements of the underlying relationship. There is extensive literature mainly in economics, microeconomics and econometrics (18, 19) are notable publications, as well as several other areas including wealth inequality, food expenditures, school quality issues and demand analysis when a more comprehensive picture of the effect of the predictors on the response variable.

Quantile regression models the relation between a set of predictor variables and specific percentiles (or quantiles) of the response variable by specifying changes in the quantiles of the response. For example, a median regression of tumor size diagnosed on brain tumor patients specifies the changes in the median tumor size as a function of the predictors. The effect of gender on the median tumor size can then be compared to the effect on other quantiles of the tumor sizes distribution and a more complete picture of covariate effects can be provided. Even more, in linear regression the regression coefficient represents the change in the response variable produced by a one unit change in the predictor variable associated with that coefficient. The quantile regression parameter estimates the change in a specified quantile of the response variable produced by a one unit change in the predictor variable. This allows comparing how some percentiles of the tumor size may be more affected by certain characteristics than other percentiles.

At various quantiles of the conditional distribution of the tumor size we are calculating coefficient estimates for the regression model shown in the equation below:

(c) $$Q\;|\;=\alpha +\beta_{1}Race\;+\beta_{2}Sex+\beta_{3}Age+\beta_{4}Age*Sex+\beta_{5}Age*Race+\beta_{6}Sex*Race+\varepsilon$$

avoiding the restrictive assumption that the error termsare identically distributed at all the points of the conditional distribution. The independent variables are Race: other, white, black; *Sex*: female, male and *Age* of the individual, *Q* is the quantile of the tumor sizes and β_1_, …, β_6_ are vectors of parameters. Our analysis includes tumor sizes measured in mm for 22, 140 individuals discussed in the result Section.

**Figure 4:**
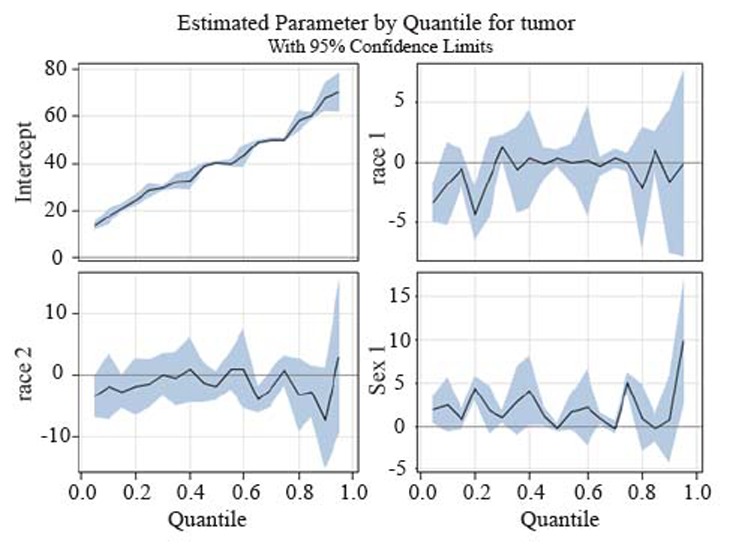
A summary of quantile regression estimates for the tumor sizes model involving three covariates and their interaction. For each of the coefficients we plot the 19 distinct quantile regression estimates. The shaded blue area depicts a 95% pointwise confidence band for the quantile regression estimates.

Figure 4 presents a summary of quantile regression results for the data. In each plot, the regression coefficients for 19 different quantiles indicate the effect on the size of the tumor with a unit change in that variable, assuming that the other variables are fixed, with 95% confidence interval bands. For example, in the first panel of the picture, the intercept can be interpreted as the estimated conditional quantile function of the tumor size distribution of a female infant whose race is “other”.

The estimated coefficients for the quantiles of the tumor sizes for the whites which are plotted in the top right graph show a negative effect on the tumor growth compared to “other race”. Regarding the effect in the median and the higher quantiles we did not see any difference with the coefficient in the linear regression case.In the bottom left graph the estimates of the quantiles regression for the blacks are not significantly different from zero, even though the effect on the 90^th^ quantile is about 14 times bigger than that on the average tumor size.In the last graph, in accordance to our OLS findings male tumor sizes are larger than females, the largest difference between the quantiles for males and females is 9.93 mm at the 95% quantile (95% CI is between 3.39 and 16.47).

In Table 6 selected results from the analysis of 10^th^, 25^th^, 50^th^, 75^th^, and 90^th^ quantiles are shown including coefficients for interaction terms between race, gender and age are tabulated to identify statistical significance with respect to the quantiles of distribution the tumor sizes, while the complete tabulation of the coefficients along with information on the statistical significance can be found on Appendix B. Age and its interaction with race appears to a statistically significant factor that affects the change in tumor size. Estimated parameter values for the variable age provide evidence that the marginal change is considerably different between mean lower and upper quantiles of tumor sizes. Specifically, estimated coefficients predict that the changes in the 5^th^, 10^th^, 15^th^, 20^th^, 25^th^, 30^th^, 35^th^quantiles of the distribution of tumor sizes are -0.06, -0.09, 0.09, -0.10, -0.12, -0.08 and -0.09 respectively for each additional year of age when the variables race and sex remain fixed were statistically significant at the 95% level. The effect of age on the conditional distribution of tumor sizes was not significant for the higher quantiles, the ordinary least squares calculation show a marginal decrease of 0.05 for each additional year.

**Table 6:** Ordinary and Quantile regression estimation of Eq. e: the coefficients on ‘tumor sizes’ reported for the 10%, 25%, 50%, 75% and 90% quantiles and the mean

	OLS	Quantile Regression				
		10	25	50	75	90

Intercept	**41.027**	**17.687**	**28.5**	**40.208**	**50.000**	**68.000**
Race 1	0.013	-1.801	-1.258	0.347	0.000	-1.615
Race 2	-0.545	-1.758	-1.3510	-1.689	0.746	-7.014
Sex 1	1.356	2.475	1.816	-0.208	**5.000**	0.768
Age	**-0.0544**	**-0.086**	**-0.125**	**-0.069**	0.000	0.000
Age*Sex 1	**0.054**	0.032	**0.081**	**0.069**	0.000	0.019
Age*Race 1	-0.003	0.054	0.045	0.000	0.000	-0.076
Age*Race 2	-0.050	0.023	-0.005	**-0.037**	0.000	-0.019
Sex1*Race1	-0.447	-0.361	0.942	-0.347	-2.000	2.905
Sex1*Race2	2.369	-0.235	1.035	**3.801**	-0.328	**8.246**

Coefficients significant at the 5% level appear in bold.

With regard to the interaction between age and race, for the 5^th^, 15^th^, 20^th^ quantiles of the distribution of the tumor sizes of the white race the effect is positive and proportional to the coefficients for a marginal change in the age while this reverses for the higher quantiles as shown in the table in the Appendix B. We also identified significant interaction between gender and age for the lower and middle quantles, the control variable being female as shown in Table 4, uniform across the quantiles and approximately equal to the estimated effect of the ordinary regression case.

Several of the covariates are of substantial public health and policy interest, the interpretation of their causal effects may be controversial, especially in the case of the gender and race covariates. In almost all the panels of Figure 4, with the exception of the coefficients for whites for the 85^th^ quantile, the quantile regression estimates do not lie at any point outside the confidence interval for the ordinary least squares regression, formal hypothesis testing is discussed in (20), suggesting that the effects of these covariates may be constant across the conditional distribution of the independent variable.

## DISCUSSION

Tumor size is known to be of great prognostic importance, independent of other prognostic variables, the purpose of this research is to conjecture on the importance of race, sex and age covariates on brain tumor size. Efforts were made to link tumor size data from registries to demographic patient information to help researchers postulate histology specific etiologic risk factors. We have applied probability and regression models to explore their impact on the size of a brain tumor. The vast majority of patients in the data set were white, the average tumor sizes diagnosed between twenty and forty years of age exhibited the largest variability, this behavior was common in both sexes. The probability distributions fitted for the tumor sizes of both male and female patients were strongly skewed, for the black female population subgroup the least amount of the variance was a result of infrequent extreme deviations from the mean. Finally, age distributions differ by histology type suggesting different etiologic factors are active for different histological types.

In addition, we explored the sources of heterogeneity in the brain tumor sizes using quantile regression to identify the effect of homogenous subpopulations associated with disease progression. The lower quantiles of the distribution of white tumor sizes are lower compared with the “other” race, whereas the 80^th^ and 90^th^ quantiles are significantly higher. When we compared the tumor sizes of blacks with other race, the estimates of the coefficients for the distribution of tumor sizes for blacks showed that the higher the quantile of the distribution of tumor sizes the bigger the difference with the relative quantile for the other races distribution. The estimated coefficients for age predict that the effect of age in the lower quantiles of the tumor size distribution is negative when the variables race and sex remain fixed.

Using the brain tumor registry data provided by SEER we demonstrated differences in tumor sizes for a histology classification. In addition, we estimated the basic characteristics of the distribution of tumor sizes as well as the significant demographical effects on them. While several approaches have been considered in the literature, we found inference with brain tumor sizes a lucid way to discuss the effect of several prognostic factors.

## ABBREVIATIONS

SEER: Surveillance Epidemiology and End Results
PDF: Probability Distribution Function
CNS: Central Nervous System
CBTRUS: Central Brain Tumor Registry of the United States
ICD: International Classification of Diseases
AYA: Adults and Young Adolescents
NOS: Non-otherwise Specified
PNET: Primitive Neuroectodermal Tumors
CI: Confidence Interval

## APPENDIX A

Formulas for the estimates of the mean, median, variance, skewness, kurtosisofthe Weibull and Dagum distributions:

Weibull:

$$\alpha \Gamma \left(1+1/\beta \right),\alpha {\left(In\,2\right)}^{1/\beta },\alpha^{2}\Gamma \left(1+2/\beta \right)-\mu^{2},\frac{\Gamma \left(1+3/\alpha \right)\beta^{3}-3\mu \sigma^{2}-\mu^{3}}{\sigma^{3}},\frac{4\Gamma \left(\frac{4}{\alpha }\right)-\frac{12}{\alpha }\Gamma \left(\frac{1}{\alpha }\right)\Gamma \left(\frac{3}{\alpha }\right)-\frac{12}{\alpha }\Gamma^{2}\left(\frac{2}{\alpha }\right)+\frac{24}{\alpha^{2}}\Gamma^{2}\left(\frac{1}{\alpha }\right)\Gamma \left(\frac{2}{\alpha }\right)-\frac{6}{\alpha^{3}}\Gamma^{4}\left(\frac{1}{\alpha }\right)}{\frac{1}{\alpha }\left[2\Gamma \left(\frac{2}{\alpha }\right)-\frac{1}{\alpha }\Gamma^{2}\left(\frac{1}{\alpha }\right)\right]}$$

Dagum:

$$-\frac{\beta }{\alpha }\frac{\Gamma \left(-\frac{1}{\alpha }\right)\Gamma \left(\frac{1}{\alpha }+k\right)}{\Gamma \left(k\right)},\beta {\left(-1+2^{\frac{1}{k}}\right)}^{\frac{-1}{\alpha }},-\frac{\beta^{2}}{\alpha^{2}}\left(2\alpha \frac{\Gamma \left(-2/\alpha \right)\Gamma \left(2/\alpha +k\right)}{\Gamma \left(k\right)}+{\left(\frac{\Gamma \left(-1/\alpha \right)\Gamma \left(1/\alpha +k\right)}{\Gamma \left(k\right)}\right)}^{2}\right),$$

$$\frac{\beta^{3}}{\sigma^{3}\Gamma^{3}\left(k\right)}\left[\Gamma^{2}\left(k\right)\Gamma \left(k+3/\alpha \right)\Gamma \left(1-3/\alpha \right)-3\Gamma \left(k\right)\Gamma \left(k+2/\alpha \right)\Gamma \left(1-2/\alpha \right)\Gamma \left(k+1/\alpha \right)\Gamma \left(1-1/\alpha \right)+2\Gamma^{3}\left(k+1/\alpha \right)\Gamma^{3}\left(1-1/\alpha \right)\right],$$

$$\frac{\beta^{4}}{\sigma^{4}\Gamma^{4}\left(k\right)}[\Gamma^{3}\left(k\right)\Gamma \left(k+4/\alpha \right)\Gamma \left(1-4/\alpha \right)-4\Gamma^{2}\left(k\right)\Gamma \left(k+3/\alpha \right)\Gamma \left(1-3/\alpha \right)\Gamma \left(k+1/\alpha \right)\Gamma \left(1-1/\alpha \right)$$

$$+6\Gamma \left(k+2/\alpha \right)\Gamma \left(1-2/\alpha \right)\Gamma \left(k\right)\Gamma^{2}\left(k+1/\alpha \right)\Gamma^{2}\left(1-1/\alpha \right)-3\Gamma^{4}\left(k+1/\alpha \right)\Gamma^{4}\left(1-1/\alpha \right)]$$

where *μ, σ^2^* are the computed means and variances and *α, β, k* as shown in Equation (b).

## APPENDIX B

**Table:**

	Intercept	Race 1	Race 2	Sex 1	Age	Age*Sex1	Age*Race1	Age*Race2	Sex1*Race1	Sex1*Race2

OLS	**41.027**	0.013	-0.545	1.356	**-0.054**	**0.054**	-0.003	-0.050	-0.447****	2.369
Quantile Regression										

5	**13.352**	**-3.352**	**-3.352**	**1.941**	**-0.058**	-0.000	**0.058**	**0.058**	0.058	-1.941
10	**17.686**	-1.801	-1.758	2.475	**-0.086**	0.032	0.054	0.020	-0.360	-0.235
15	**21.216**	-0.489	**-2.640**	0.783	**-0.093**	**0.060**	**0.033**	0.033	**-1.510**	0.640
20	**24.200**	**-4.200**	-1.765	**4.400**	**-0.100**	0.000	**0.100**	0.013	0.600	1.339
25	**28.500**	-1.258	-1.351	1.816	**-0.125**	**0.080**	0.044	-0.004	0.942	1.034
30	**29.257**	**1.219**	0.084	**1.092**	**-0.085**	**0.079**	0.006	0.036	**-1.568**	1.227
35	**32.454**	-0.608	-0.560	2.874	**-0.091**	**0.051**	0.039	-0.037	-1.720	0.767
40	**32.83**	0.298	0.876	4.164	-0.044	**0.044**	0.000	-0.071	-1.298	0.338
45	**38.843**	-0.093	-1.266	1.156	**-0.093**	**0.093**	0.000	-0.038	0.093	1.574
50	**40.208**	0.347	-1.689	-0.208	**-0.069**	**0.069**	-0.000	-0.037	-0.347	3.800
55	**40.000**	0.000	0.857	1.686	-0.000	0.019	-0.000	**-0.107**	0.254	4.069
60	**43.058**	0.150	1.032	2.141	-0.035	**0.071**	-0.006	-0.078	-0.409	1.579
65	**49.073**	-0.341	**-3.489**	**0.926**	**-0.073**	**0.073**	-0.000	-0.001	0.341	**3.913**
70	**50.142**	0.311	-2.169	-0.192	**-0.017**	**0.030**	-0.012	-0.049	-0.261	**4.775**
75	**50.000**	0.000	0.746	**5.000**	-0.000	0.000	-0.000	-0.029	**-2.000**	-0.328
80	**58.106**	-2.160	-3.224	0.968	0.053	**0.067**	-0.014	-0.029	3.085	4.164
85	**60.000**	0.968	-2.652	-0.187	0.000	**0.031**	**-0.031**	**0.087**	-0.781	**6.126**
90	**68.000**	-1.615	-7.014	0.768	0.000	0.019	-0.076	-0.019	2.905	**8.246**
95	**70.000**	-0.071	3.142	**9.928**	0.035	-0.035	-0.035	-0.107	-3.607	0.928
